# Anti-Metastasis Effect of Fucoidan from *Undaria pinnatifida* Sporophylls in Mouse Hepatocarcinoma Hca-F Cells

**DOI:** 10.1371/journal.pone.0106071

**Published:** 2014-08-27

**Authors:** Peisheng Wang, Zhichao Liu, Xianli Liu, Hongming Teng, Cuili Zhang, Lin Hou, Xiangyang Zou

**Affiliations:** 1 Department of Biotechnology, Dalian Medical University, Dalian, PR China; 2 College of Life Sciences, Liaoning Normal University, Dalian, PR China; University of South Alabama Mitchell Cancer Institute, United States of America

## Abstract

Metastasis is one of the major causes of cancer-related death. It is a complex biological process involving multiple genes, steps, and phases. It is also closely connected to many biological activities of cancer cells, such as growth, invasion, adhesion, hematogenous metastasis, and lymphatic metastasis. Fucoidan derived from *Undaria pinnatifida* sporophylls (Ups-fucoidan) is a sulfated polysaccharide with more biological activities than other fucoidans. However, there is no information on the effects of Ups-fucoidan on tumor invasion and metastasis. We used the mouse hepatocarcinoma Hca-F cell line, which has high invasive and lymphatic metastasis potential *in vitro* and *in vivo*, to examine the effect of Ups-fucoidan on cancer cell invasion and metastasis. Ups-fucoidan exerted a concentration- and time-dependent inhibitory effect on tumor metastasis *in vivo* and inhibited Hca-F cell growth, migration, invasion, and adhesion capabilities *in vitro*. Ups-fucoidan inhibited growth and metastasis by downregulating vascular endothelial growth factor (VEGF) C/VEGF receptor 3, hepatocyte growth factor/c-MET, cyclin D1, cyclin-dependent kinase 4, phosphorylated (p) phosphoinositide 3-kinase, p-Akt, p-extracellular signal regulated kinase (ERK) 1/2, and nuclear transcription factor-κB (NF-κB), and suppressed adhesion and invasion by downregulating L-Selectin, and upregulating protein levels of tissue inhibitor of metalloproteinases (TIMPs). The results suggest that Ups-fucoidan suppresses Hca-F cell growth, adhesion, invasion, and metastasis capabilities and that these functions are mediated through the mechanism involving inactivation of the NF-κB pathway mediated by PI3K/Akt and ERK signaling pathways.

## Introduction

Tumor metastasis is a multistep process by which cancer cells from the primary tumor enter the vasculature and circulate, migrate, and invade to distant secondary organs or tissues. The processes of the tumor metastatic cascade require the survival, growth, invasion, and signal transduction potential of tumor cells. Cyclin D1 and cyclin-dependent kinase 4 (CDK4) are key players in the cell cycle and growth [1]. L-Selectin belongs to the selectin family, which is related to tumor metastasis, adhesion, and lymphocyte homing [2]. Matrix metalloproteinases (MMPs) and their specific inhibitors–tissue inhibitor of metalloproteinases (TIMPs) have been acknowledged as major critical molecules associated with extracellular matrix (ECM) degradation and cancer cell invasion during metastasis [3]–[6]. Vascular endothelial growth factor C (VEGF-C) and VEGF receptor 3 (VEGFR-3) are potent lymphatic endothelial cell (LEC) molecules involved in growth and are key regulators of lymphangiogenesis [7]. Moreover, some researchers have reported that the proliferative actions of VEGF require activation of both the extracellular signal–regulated kinase (ERK) and Akt signaling cascades [8]–[10]. Similarly, hepatocyte growth factor (HGF) and c-MET play important roles in the control of tumor growth, invasion and metastasis [11]–[13]. It is also well established that nuclear transcription factor-κB (NF-κB) is most frequently involved in cell survival and growth through the transactivation of anti-apoptotic genes [14]. However, NF-κB activity is connected with multiple signaling pathways, such as the phosphoinositide 3-kinase (PI3K)/Akt and ERK signaling pathways. These pathways play a pivotal role in cancer formation and progression by modulating cell growth, apoptosis, and metastasis [15], [16].

Fucoidan is a highly sulfated polysaccharide of brown algae whose main sugar unit is sulfated fucose [17]. It has been reported to have a wide range of physiological and biological activities, such as anti-inflammatory, anticoagulant, anti-tumor, anti-metastatic, and antiangiogenesis activities [18]–[20]. The biological activities and medicinal effect of fucoidan depend greatly on its structural properties, molecular weight, sulfate content, etc. Fucoidans from different algal species and extraction techniques differ from each other [21]–[24]. Fucoidan extracted from sporophylls of the brown seaweed *Undaria pinnatifida* (Ups-fucoidan) has a higher sulfate and L-fucose content and a wider range of physiological and biological activities than other fucoidans [24]. The anti-tumor activity of Ups-fucoidan has garnered much attention. Previously, we demonstrated that Ups-fucoidan induced apoptosis in SMMC-7721 cells via the reactive oxygen species–mediated mitochondrial pathway [25] and inhibited angiogenesis by downregulating VEGF-A expression in human umbilical vein endothelial cells [26].

In the present study, we evaluated the effects of Ups-fucoidan on tumor metastasis of Hca-F cells. The study comprised four experiments: tumor growth, adhesion, invasion, and metastasis. Our findings will aid understanding of the anti-tumor and anti-metastasis effects of Ups-fucoidan and the mechanism of anti-metastasis.

## Materials and Methods

### Ethics Statement

All studies involving mice were approved by the Dalian Medical University Animal Care and Use Committee (Approval No. 2008-0002). All surgeries were performed under general anesthesia and efforts were made to minimize suffering to animals.

### Purification and Analyses of Ups-fucoidan

Ups-fucoidan was extracted routinely by treating *U. pinnatifida* sporophylls with hot water extraction and alcohol grade precipitation, DEAE-cellulose, and Sephadex G-100 column chromatography. Infrared spectra and ^13^C-NMR spectra were detected using Nicolet 510P (Thermo Fisher Scientific) and Bruker AV-500 NMR spectrophotometers (Bruker Optik GmbH, Ettlingen, Germany), respectively. Proteins, total carbohydrates, sulfate radicals, and uronic acid were measured by the bicinchoninic (BCA) protein assay (KeyGen Biotech, Nanjing, China), phenol-sulfuric acid reaction, BaCl_2_/gelation, and sulfuric acid–carbazole colorimetric method, respectively. The purify and analyze method was performed as previously described [25], [26]. The molecular weight of the sample was determined by size exclusion chromatography using TSK-gel G3000PWXL (Tosoh, Tokyo, Japan). The optical rotation of the sample was measured by a WZZ-1 polarimeter (INESA.CC, Shanghai, China).

### Cell Culture

The mouse Hca-F hepatocarcinoma cell line (established and stored by Department of Pathology, Dalian Medical University, Dalian) has high invasive and lymphatic metastasis potential [27]. The cells were maintained in 90% RPMI 1640 medium (Thermo Fisher Scientific, CA, USA) supplemented with 10% FBS (Thermo Fisher Scientific), penicillin (100 U/ml), and streptomycin (100 µg/ml) (Thermo Fisher Scientific) at 37°C in a humidified incubator containing 5% CO_2_.

### Cell Growth Assay

Cell growth was measured by the MTT method. Briefly, logarithmic-phase growing Hca-F cells were harvested (survival rate>99%) and seeded in 96-well plates (5.0×10^4^ cells/well, in 100 µl medium). The cells were treated with Ups-fucoidan (0, 250, 500, or 1000 µg/ml) in a final volumes of 200 µl for 6, 12, 24, 48, or 72 h. Twenty microliter MTT (5 mg/ml dissolved in PBS; Sigma-Aldrich, CA, USA) was added to each well 4 h prior, and the plates were reincubated for another 4 h. The formazan product was dissolved in 150 µl DMSO and the absorbance (*A*) of each plate was measured at 492 nm using a Multiskan Ascent microplate photometer (Thermo Fisher Scientific). Untreated cells were used as the control. The inhibition rate (*I*%) was calculated using the following equation: *I* (%)* = *[1−(*A*_treated_−*A*_blank_)/(*A*_control_−*A*_blank_)]×100%; Similarly, cell death was identified by trypan blue staining, after the treatment of different concentrations of Ups-fucoidan for 24 h. The number death cells and total cells were counted. Counting the cell death rate (*D*%) using the following equation:

*D*% = (*C_d_*_eath cell_ */C*_total cell_)×100%, all calculations were conducted in triplicate.

### Cell Adhesion Assay

Cells were cultured in fresh RPMI 1640 complete medium containing Ups-fucoidan (0, 250, 500, or 1000 µg/ml) at a final density of 5×10^6^ cells/ml. The cells were overlaid on a frozen lymph node section and the sample was incubated with 5% CO_2_ at 37°C for 16 h. After 30-min static incubation at 4°C, the sample was fixed in 95% alcohol for 5 min, followed by hematoxylin and eosin (HE) staining and undergoing an *in vitro* adhesion assay as previously described [28]. Adherent cells were counted under a light microscope and expressed as the average of five fields.

### Cell Invasion Assay

The cell invasion assay was performed as previously described [29], [30] using Matrigel (Sigma-Aldrich, CA, USA) (diluted in serum-free RPMI 1640 medium, 1∶4) pre-coated Transwell chamber inserts (diameter: 6.5 mm; pore size: 8 µm; Corning, NY, USA). Cells were cultured in serum-free RPMI 1640 medium containing Ups-fucoidan (0, 250, 500, or 1000 µg/ml) at a final density of 5×10^4^ cells/ml. Cell suspensions (200 µl) from each test group were added to the top chamber, and 500 µl fresh RPMI 1640 medium containing 10% FBS was placed in the bottom chamber. The Transwell chambers were incubated for 24 h at 37°C. The cells on the upper surface of the insert were removed by swabbing. Cells that had invaded were fixed and stained with absolute methanol for 10 min and then with 0.1% crystal violet for 30 min. Cell invasion was quantified by counting the cells that had invaded to the bottom side of the filter under a light microscope (×200 magnification). Five fields were counted for each test group to obtain the average. Experiments were performed thrice in triplicate.

### Western Blot Analysis

The expression levels of the relevant genes were evaluated by western blotting. Hca-F cells (3.0×10^6^ cells/well, 2 ml) seeded in 6-well plates were treated with Ups-fucoidan (0, 250, 500, or 1000 µg/ml) for 24 h. The culture medium was collected for ELISA. Cells were lysed in 100 µl RIPA lysis and extraction buffer (Thermo scientific pierce). Total protein was determined using the BCA protein assay. Equal amounts of protein were loaded and fractionated by 12% SDS-PAGE and transferred onto nitrocellulose (NC) membranes (Solarbio Science & Technology, Beijing, China). The NC membranes were incubated with rabbit polyclonal primary antibodies against VEGFR-3 (1∶1500; Santa Cruz Biotechnology, CA, USA), and reacted with goat anti-rabbit horseradish peroxidase (HRP)-conjugated secondary antibodies (1∶5000; Thermo Fisher Scientific). Blots were detected with a Bio-Rad imaging system (CA, USA). The graphs were analyzed using the image gray scale analysis method using Gel-pro analyzer software.

The western blot steps of other proteins were similar to VEGFR-3, the dilution conditions as follows: Rabbit anti-Cyclin D1 (1∶500), CDK4 (1∶500), Akt (1∶1000), TIMP-3 (1∶1000), c-MET (1∶500) and antibodies were purchased from Sangon Biotech Co. (Nanjing, China), TIMP-1 (1∶500) and L-Selectin (1∶1000) antibodies were purchased from Solarbio Science & Technology (Beijing, China), β-Actin (1∶1000; KeyGen Biotech, Nanjing, China), Mouse anti-GAPDH (1∶10000; KangChen Bio-tech, Shanghai, China), rabbit anti-mouse immunoglobulin G (H+L) secondary antibodies (1∶5000) were supplied by Thermo Fisher Scientific, and other antibodies were purchased from Bioworld Technology (Nanjing, China), such as NF-κB (1∶500), p-PI3K (1∶500), p-Akt (1∶500), ERK1/2 (1∶1000), p-ERK1/2 (1∶1000).

### ELISA

VEGF-C and HGF levels were evaluated by ELISA in 96-well plates coated with polyclonal anti–VEGF-C (1∶1000), anti-HGF (1∶100) antibodies diluted in 50 mM carbonate buffer (pH 9.0), respectively. Blocking buffer (PBS containing 1% BSA and 0.02% azide) was added to block non-specific protein binding. Different concentrations of VEGF-C and HGF growth factors were used to draw standard curve, respectively. Culture medium samples collected from the western blot assay were diluted 1∶100 in blocking buffer were added. The plates were washed with PBS containing 0.05% Tween-20. Rabbit anti-mouse HRP-conjugated secondary antibody (1∶5000 in blocking buffer) was added, respectively. Tetramethylbenzidine HRP color development solution (KeyGen Biotech) was added for 10 min development in a darkroom. The reaction was stopped by adding 50 µl 2 M H_2_SO_4_. The plates were read on a Multiskan Ascent photometer (Thermo Fisher Scientific) at *A*450.

### *In Vivo* Tumor Metastasis Assay

Eight-week-old male 615 mice (specific pathogen-free) were obtained from the Dalian Medical University Experimental Animal Center. *In vivo* tumor metastasis assay was performed as previously described [31]. Twenty-four 615 mice were equally assigned to four groups. Hca-F cells (3×10^6^, 30 µl) were inoculated subcutaneously into the footpads of the mice. After 48 h, normal saline (NS, control group, 30 µl) and Ups-fucoidan (treated groups, 125, 250 mg/kg, 30 µl), or heparin (positive group, 125 µg/kg, 30 µl) was injected into the same footpads once every other day. After four weeks, the axillary lymph nodes were isolated, weighed, sectioned, and stained with HE.

### Statistical Analysis

Statistical analysis was performed using SPSS v17.0. Each experiment was carried out twice with triplicate measurements for quantitative comparison; data are expressed as the mean ± SD. One-way ANOVA was used to determine the significance of the differences in multiple comparisons; *p*<0.05 and *p*<0.01 were considered statistically significant.

## Results

### Preparation and properties of Ups-fucoidan

The physical and chemical characters of Ups-fucoidan have been described [26]. The Ups-fucoidan was a beige fibrous powder (purity>90%). It contained 0.13% protein, 68.37% carbohydrate, 21% sulfates and 10.89% uronic acid, and had a molecular weight of 10.4356×10^4^ Da. The optical rotation of the sample (0.6 mg/ml, 20°C) showed a value of 0.99° at 589 nm.

### Ups-fucoidan Inhibited Hca-F Cell Growth *In Vitro*

**Figure 1:**
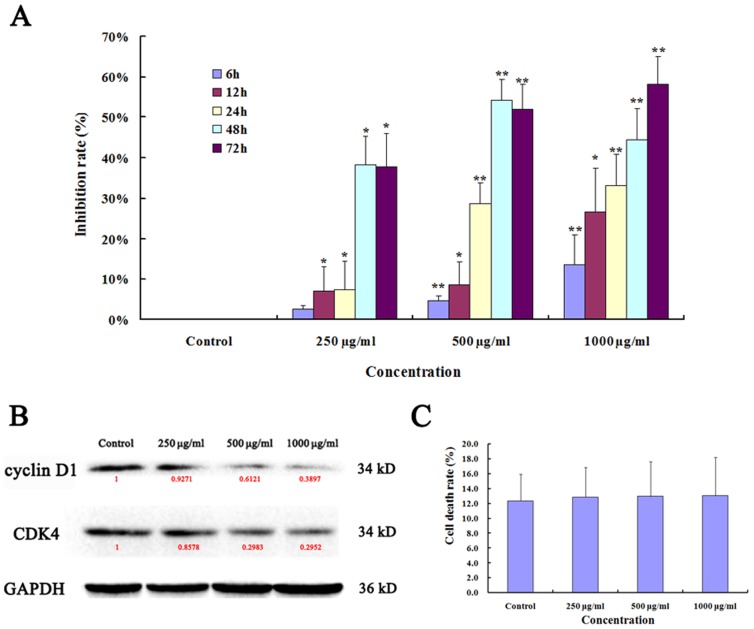
Ups-fucoidan inhibits Hca-F cell proliferation. (A) Concentration- and time-dependent effect of Ups-fucoidan on Hca-F cell growth *in vitro* measured with MTT assay. (B) Cyclin D1 and CDK4 expression were significantly decreased following Ups-fucoidan treatment. Values in red denote the relative expression quantity of each protein compare with GAPDH, respectively. (C) The cell death rate (*D*%) was counted and calculated by trypan blue staining. There is not statistically significant between Ups-fucoidan treatment and control group. Data are the mean ± SD from three independent experiments. *p<0.05 and **p<0.01 compared with untreated control (One-way ANOVA).

The effects of 0, 250, 500, or 1000 µg/ml Ups-fucoidan on Hca-F cell growth were assessed in vitro via measurement of cell viability by MTT assay. Cell growth following each Ups-fucoidan treatment was statistically significant compared with that of the untreated control (Figure 1A). In western blotting, Ups-fucoidan downregulated the expression levels of both cyclin D1 and CDK4 (Figure 1B). After Ups-fucoidan withdrawal, counting the death cell by trypan blue staining and calculating the cell death rate (*D*%), and all the *D*% in each concentrations group of Ups-fucoidan is not statistically significant compared with control. These findings indicate that Ups-fucoidan could inhibit Hca-F cells growth, but not cyto-toxicity.

### Ups-fucoidan Inhibited Hca-F Cell Adhesion *In Vitro*

**Figure 2:**
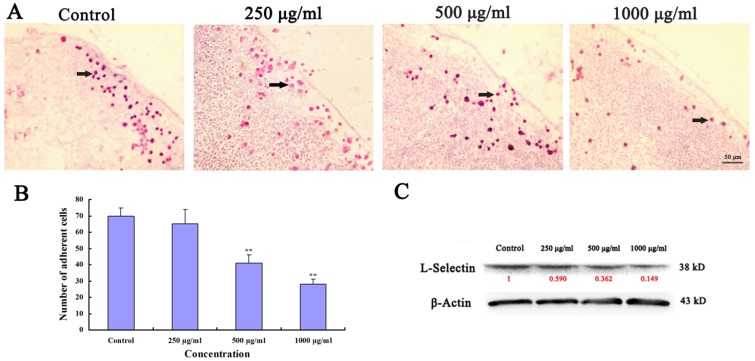
Ups-fucoidan inhibits Hca-F cell adhesion. (A) Binding of Hca-F cells to lymphatic endothelium measured *in vitro* by adhesion assay. (B) Adherent cells expressed as the average of five fields. There were fewer (*p*<0.01) adherent Ups-fucoidan–treated cells than control cells. (C) Western blot of downregulated L-Selectin expression following Ups-fucoidan treatment. Values in red denote the relative expression quantity of the L-Selectin expression compare with β-Actin, respectively. Data are the mean ± SD from three independent experiments. **p<0.01 compared with untreated control (One-way ANOVA).

The adhesion assay was used to measure the binding of Hca-F cells to peripheral lymphatic endothelium. The adhesion capability of Hca-F cells treated with 500 µg/ml (41±5.12 cells/field; *p*<0.01) and 1000 µg/ml (28±3.13 cells/field; *p*<0.01) Ups-fucoidan (Figure 2A) were significantly lower than that of the control (70±5.01 cells/field) (Figure 2B). L-Selectin, as adhesion molecule, plays important role in tumor adhesion to lymph nodes. Generally, L-Selectin is specifically expressed in leukocytes, but also in tumor cells, which having high potential of lymphatic metastasis. The findings indicate that Ups-fucoidan down regulated the expression of L-Selectin, which inhibited the capability of Hca-F cells to adhere to peripheral lymphatic endothelium.

### Ups-fucoidan Inhibited Hca-F Cell Invasion *In Vitro*

**Figure 3:**
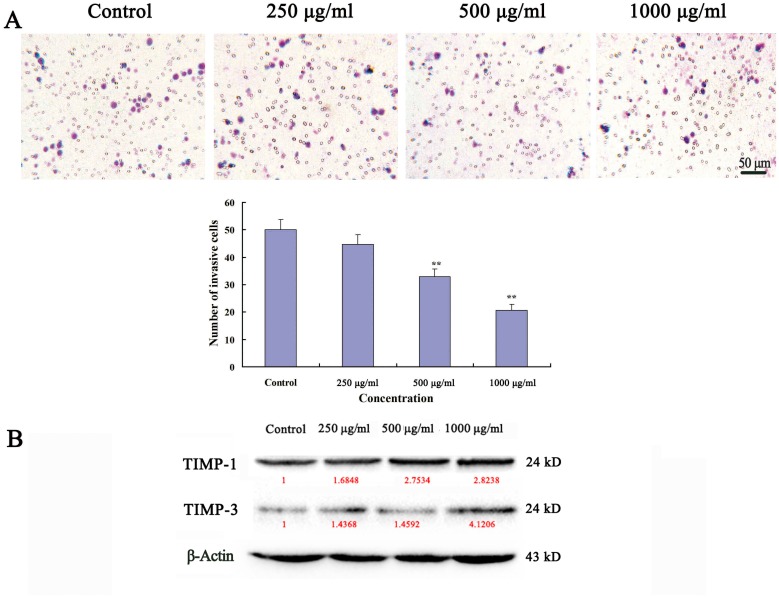
Ups-fucoidan inhibits Hca-F cell invasion. (A) The dark blue appearance cells that invaded through the Matrigel membrane were stained and counted. Values expressed are the average of five fields. (B) Western blot results showing that Ups-fucoidan increased TIMP-1 and TIMP-3 expression in a concentration-dependent manner. Values in red denote the relative expression quantity of each protein compare with GAPDH, respectively. Data are the mean ± SD of three independent experiments. **p<0.01 compared to the untreated control indicates statistical significance (One-way ANOVA).

In the invasion assay, Ups-fucoidan suppressed Hca-F cell invasion *in vitro*. The number of Ups-fucoidan–treated cells (1000 µg/ml, 24 h; 21±2.19 cells/field) that invaded through the Matrigel-coated filter was significantly lower compared with that of the control (50±3.81 cells/field; *p*<0.01) (Figure 3A). Western blot were used to evaluate the relative expression quantity of TIMP-1 and TIMP-3. The expression of TIMP-1 and TIMP-3 expressions were higher compared to the control (Figure 3B).

### Ups-fucoidan Modulated Inactivation of Related Signaling Pathways

**Figure 4:**
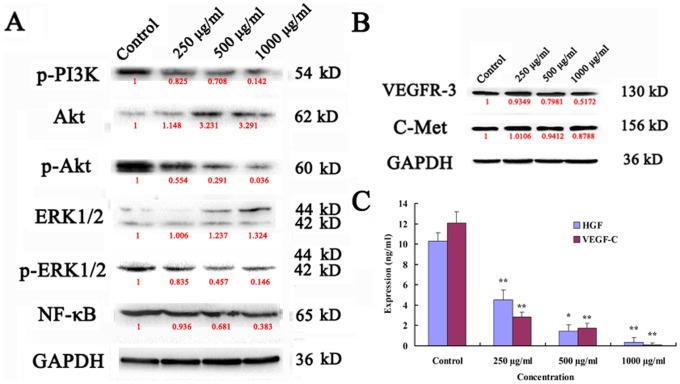
Western blot and ELISA were used to evaluate the inactivation of related signaling pathways. (A), (B) Western blots results showed that the expression of VEGFR-3, c-MET, p-PI3K, p-Akt, p-ERK1/2, and NF-κB were downregulated following Ups-fucoidan treatment, and ERK1/2 was upregulated. Values in red denote the relative expression quantity of the each protein compare with GAPDH, respectively. (C) ELISA demonstrating significantly lower the expression quantity of VEGF-C and HGF following Ups-fucoidan treatment compared to the control. Data are the mean ± SD from three independent experiments. *p<0.05 and **p<0.01 compared with control (One-way ANOVA).

Western blotting and ELISA were used to evaluate the activity of the NF-κB–dependent PI3K/Akt and ERK signaling pathways and the related key factors. VEGFR-3, c-MET, p-PI3K, p-Akt, p-ERK1/2, and NF-κB expression were downregulated in Ups-fucoidan–treated cells (Figure 4A, B). ELISA results showed that the levels of VEGF-C and HGF protein in Ups-fucoidan–treated cells were lower (*p*<0.01) compared to the control (Figure 4C).

### Ups-fucoidan Inhibited Hca-F Cell Metastasis *In Vivo*

**Figure 5:**
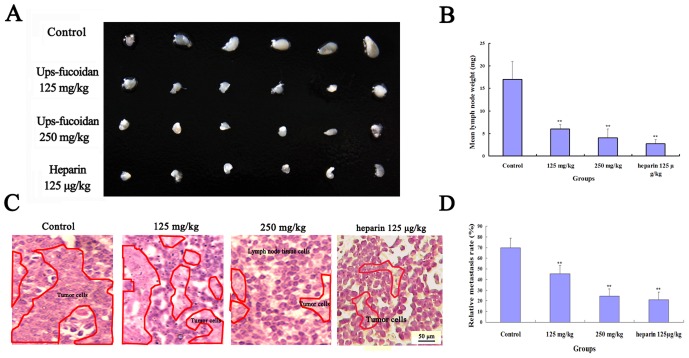
Ups-fucoidan inhibits tumor metastasis to peripheral lymph nodes *in vivo*. Twenty-four 615 mice were equally assigned to four groups. (A) Axillary lymph nodes from mice treated with heparin, NS or Ups-fucoidan. (B) Average weights of the axillary lymph nodes from mice in each test group. (C) Axillary lymph nodes from mice treated with NS or Ups-fucoidan following inoculation with Hca-F cells (H.E staining). Notes indicate that the tissue structures of lymphatic metastasis of Hca-F cells were obscure, however, the normal lymphocyte were obvious and the cell nucleus were stained by blue (×400 magnification). (D) Relative metastasis rate was calculated by counted the metastatic Hca-F cells and normal lymphocyte. There were fewer (*p*<0.01) metastatic Hca-F cells in treated groups than in control group. **p<0.01 compared with control (One-way ANOVA).

The mean lymph node metastasis weight in 615 mice inoculated with Hca-F cells was lower (*p*<0.01) when the inoculation was followed by Ups-fucoidan treatment compared to that followed by NS (control); Figure 5A and B depicted the axillary lymph nodes of the Ups-fucoidan groups. The average weight of the axillary lymph nodes of Ups-fucoidan–treated mice were also significantly lower (*p*<0.01) compared to those of the control. Heparin, as positive control, showed obvious inhibitory effect in reducing lymph node metastasis, but also has serious hemorrhagic tendency. Histological analysis showed that Ups-fucoidan attenuated Hca-F cell infiltration into the lymph nodes (Figure 5C and D). The results indicate that Ups-fucoidan could inhibit tumor metastasis to the peripheral lymph nodes *in vivo*.

## Discussion

Metastasis, where cancer cells spread from the primary site to distant organs, is the leading cause of death from cancer. The metastatic processes involving the vascular and lymphatic systems have been acknowledged. In addition, metastasis via the lymphatic system precedes metastasis via the vascular system in many cancers [32].

Fucoidan isolated from *U. pinnatifida* has a higher sulfate and L-fucose content and more bioactivities [33]–[38] than that extracted from other brown seaweeds. It has also become the center of much attention because of its anti-cancer activity and is expected to be a new candidate for low-toxicity cancer therapy [39], [40]. The Ups-fucoidan we extracted from the *U. pinnatifida* sporophylls had higher sulfate content and better activities than that from other *U. pinnatifida* segments [24], [41]. During lymphatic system metastasis, tumor cells activate related signal molecules, which are related with tumor growth, ECM degradation, cancer cell invasion and metastasis. Unfortunately, the mechanisms of Ups-fucoidan anti-metastasis activity have not been elucidated. Therefore, we evaluated Ups-fucoidan bioactivities and investigated their linked mechanisms. We found that the pharmacological actions of Ups-fucoidan were associated with inhibition of NF-κB–dependent PI3K/Akt and ERK pathway activation, which regulate inactivation of the VEGF-C/VEGFR-3, HGF/c-MET dual signaling pathways.

The cyclin D1–CDK4 axis is a key player in tumor growth and development. Deregulation of the cyclin D1–CDK4 axis is a common feature in malignant tumor cells [42]. L-Selectin acts as a cell adhesion molecule and signaling receptor, contributing to regulate traffic of leukocytes to lymph nodes and playing an important role in tumor adhesion and transmigration [43]. Generally, L-Selectin is specifically expressed in leukocytes, but also in tumor cells, which having high potential of lymphatic metastasis. Our results indicate that Ups-fucoidan down-regulated the expression of L-Selectin in a concentration-dependent manner. MMPs are considered major critical molecules that assist tumor cells during metastasis, and TIMPs modulate MMPs activation, and suppressed the degradation of ECM [5]. NF-κB activation may contribute to cellular resistance to growth and invasion by upregulating the genes involved in cell survival, cell–cell adhesion, and cell–ECM interaction [44]. We speculated that inhibition of NF-κB activity may suppress Hca-F cell growth and invasion. In the present study, Ups-fucoidan suppressed Hca-F cell growth, adhesion, and invasion by downregulating cyclin D1–CDK4, L-Selectin, upregulating TIMP-1 and TIMP-3, which are regulated by the NF-κB pathway.

In addition, NF-κB inactivation is related to the PI3K/Akt and ERK signaling pathways [35]. Hepatocarcinoma tumor cells release HGF, which stimulates MET activity, and MET activation in cell amplification promotes activation of PI3K/Akt and ERK signaling [45], [46]. Interestingly, HGF/MET signaling has also been reported to promote lymphangiogenesis and endothelial cell growth through interaction with the VEGF and VEGFR pathways [47]. VEGFs are commonly overexpressed in several cancers. VEGF-C is a representative member of the VEGF family and can bind with its cognate receptor VEGFR-3, stimulating lymphangiogenesis and new growth of lymphatic vessels. Moreover, VEGF-C expression according to tumor cell dose correlated with growth and metastatic potential *in vitro* and *in vivo* [48]–[50]. VEGFR-3 is a tyrosine kinase receptor expressed primarily on LECs. Researchers have proved that lower VEGFR-3 expression correlates with fewer positive lymph nodes and longer survival [51]. We believe it is noteworthy that we are the first to observe VEGFR-3 expression in Hca-F cells and that Ups-fucoidan treatment decreased it. This finding indicates that the HGF/MET and VEGF-C/VEGFR-3 dual signaling pathways may be the reason the Hca-F cell line has high lymphatic metastasis potential. In our study, VEGF-C/VEGFR-3, HGF/c-MET, p-PI3K, p-Akt, p-ERK1/2, and NF-κB expression were downregulated in Ups-fucoidan–treated cells. The results indicate that Ups-fucoidan inhibits tumor growth and metastasis by inactivating the related signaling pathways.

Recent studies demonstrate Ups-fucoidan to be a potential preventive or therapeutic agent for treating cancers. The antitumor effects of Ups-fucoidan may be connection with the inhibition of tumorigenesis and metastasis. However, the specific mechanisms of the anticancer effects of Ups-fucoidan have not been fully investigated. Therefore, the antitumor effect and the relationship between bioactivity and structural characteristics of Ups-fucoidan will be significative for further investigation and using as a marine drug.

## Conclusion

Taken together, we have shown that the effect of Ups-fucoidan suppression of tumor growth, adhesion, invasion and metastasis of mouse hepatoma Hca-F cells through PI3K/Akt and ERK signaling pathways. Our findings indicate that Ups-fucoidan could have a potential for inhibiting tumor metastasis.
